# Supplementary material for: Chromothripsis during telomere crisis is independent of NHEJ, and consistent with a replicative origin
Source: Genome Res. 2019 May;29(5):737–49. doi: 10.1101/gr.240705.118 (PMC6499312; doi:10.1101/gr.240705.118)
Supplement: Supplemental Material [file supp_gr.240705.118_Supplemental_file_1.zip › contigs/annotated_contigs/DB53/contig.2.DB53_length_480_mean_cov_5.56458333333.docx]

**DB53_length_480_mean_cov_5.56458333333**

```
AACCTAAGTTTAGTTTTGACTATCT|TTTTCTTAATAGCTTTGGGTAAATAATTTATTGTTTCTATCTTAAACAATGGTCCCTTGCCTT
                          >chr8:125426073-125426311 + E=1e-125
TGCATAACTTTCAGTACATAAGTTTGAATGTAATTTATAAAATTGAGATGGTTATTAATAAGAATAAAGAGATAAGGTGTTTTCTCAGA

AAGCATGCATAATTAATATTATTGCCTTAATGATTAAATTGTACTTAATGAACCATATTCTGCCAGAGTCTGAAGTAGTCCCTTGT|CT

CTGTTACCCATGCTGTAGTGCAGTGGTGTGATCTTGGCTTACGGCAACCTCCGCCTCCCAGGTTCAAGTGATTCTCTCTCCTCAGCCTC
>chr7:155691334-155691551 + E=5e-113
CTAAGTAGCTGGGATTACAGATGCGTGCCACTACAGCCCTGCTAATTTTTGTATTTTTAGTAGAGACAGGGTTTCACCATGTTGGCCAG

GTTGGTCTCAAACTCAGGTGATTGGCCTGCCTTGGCC
```
